# Impact of the COVID-19 pandemic on early clinical outcome after total knee arthroplasty: a retrospective comparative analysis

**DOI:** 10.1007/s00402-022-04597-w

**Published:** 2022-09-01

**Authors:** Patrick Reinbacher, Ulrike Wittig, Georg Hauer, Alexander Draschl, Andreas Leithner, Patrick Sadoghi

**Affiliations:** Department of Orthopaedics and Trauma, Medical University of Graz, Auenbruggerplatz 5, 8036 Graz, Austria

**Keywords:** Total knee arthroplasty, COVID-19, Physiotherapy, Postoperative care, Rehabilitation

## Abstract

**Introduction:**

To help combat the SARS-CoV-2 (COVID-19) pandemic, elective inpatient procedures have been reduced. The authors hypothesized that a nationwide lockdown would negatively affect the postoperative outcome after total knee arthroplasty (TKA) due to reduced physiotherapy as well as restrictions in external facilities of physiotherapy and rehabilitation.

**Materials and methods:**

We conducted a retrospective, comparative study including 41 patients who had undergone primary TKA during the first lockdown of the COVID-19 pandemic from March 2020 to April 2020 and a comparable control group consisting of 47 patients with a minimum follow-up of 6 months before the COVID-19 pandemic from 2019. Relevant end points were the visual analogue scale (VAS) for pain, Knee Society Function Score (KSS), Oxford Knee Score (OKS), Western Ontario and McMaster Universities Osteoarthritis Index (WOMAC), and range of motion (ROM).

**Results:**

The lockdown group had a significantly worse outcome compared to the control group 6 months after TKA regarding WOMAC (*p* = 0.001), KSS (*p* < 0.001), OKS (*p* < 0.001), and length of hospital stay (*p* < 0.001). We found no statistically significant difference between the groups in ROM (*p* = 0.132), KSFS (*p* = 0.933), VAS at rest (*p* = 0.9.22), and exercise (*p* = 0.304).

**Conclusion:**

The COVID-19 pandemic negatively affected early clinical outcome parameters of elective primary TKA at 6 months of follow-up due to restrictions in postoperative care. We believe that standardized protocols for physiotherapy will improve clinical outcomes for TKA in the event of future lockdowns and underline the importance of appropriate postoperative care during this pandemic.

## Introduction

During the first wave of the pandemic, elective inpatient procedures wer reduced in many European hospitals to combat SARS-CoV-2 (COVID-19). Elective surgeries that would have required postoperative management in the intensive care unit were postponed entirely. These initial efforts were made to ensure sufficient capacities for acute trauma care and COVID patients [1, 2].

Such drastic efforts were important steps in the worldwide fight against the COVID-19 pandemic. However, the restrictions strongly affected public orthopaedic departments, as many operations are elective and therefore designated as non-essential. Although daily evaluation of the pandemic situation by a task force from the hospital enabled a flexible adaptation to the current circumstances, the reduction in the number of operating rooms limited the daily capacity of surgical therapy. There was also a clear recommendation for orthopaedic surgeons to reduce patient crowding in outpatient clinics [3–5].

Consequently, patients who underwent elective TKA during the first lockdown faced new challenges, especially in terms of physiotherapy. The therapy programme of postoperative physiotherapy was reduced to a minimum, and local hygiene regulations were implemented to limit the spread of COVID-19 [1, 3, 26]. From 20th of March onwards, the lockdown restrictions in the central European countries were extended, and external facilities for physiotherapy and rehabilitation were closed until the 30th of April, except for the use of necessary medical rehabilitation measures following acute medical treatment [6]. The lockdown restrictions became obsolete on the 1st of May.

The restrictions in physiotherapy and closures of external rehabilitation facilities imposed by the COVID-19 pandemic are unique in the history of modern medicine [7]. Hence, no validated historical and current data are available to predict the impact of a national lockdown on the healthcare system, especially on elective orthopaedic surgeries and their clinical outcomes [7]. So far, only one recent study has examined the impact of the pandemic on elective orthopaedic surgeries such as arthroplasty and spinal fusion in the USA based on a stochastic simulation [7].

The current literature comprises studies that investigated the impact of the pandemic on different aspects of orthopaedic care. These include handling orthopaedic disease triage, managing orthopaedic surgery during the pandemic, the return after the lockdown period, and the postoperative monitoring of orthopaedic patients [8–10]. However, literature lacks sufficient data regarding the impact of the COVID-19 pandemic on orthopaedic surgery as measured by clinical outcome scores after total knee arthroplasty (TKA).

It is evident that adequate physiotherapy after TKA is essential for an enhanced clinical outcome [7, 11]. This study provides further insight into this evaluation, as no trial has yet been conducted to evaluate the early postoperative outcome after TKA during the first wave of the pandemic. Therefore, the purpose of this study was to evaluate the impact of the COVID-19 pandemic on clinical outcomes after TKA regarding clinical parameters. The study hypothesis was that restrictions on postoperative care due to the pandemic would negatively affect the functional outcome of patients following TKA.

## Methods

### Trial design

A retrospective comparative analysis of two cohorts was conducted in a single, urban, high-volume university hospital in Austria. The demographic and baseline characteristics of the two groups are summarized in Table 1.

**Table 1:** Patient demographics and baseline characteristics

Demographics	COVID (*n* = 41)	Non-COVID (*n* = 47)	*p *value
Age (years), mean (SD)	65.2 (10.5)	70.8 (7.9)	0.006
Sex (M/F) (*n*/%)	12(29%)/29(71%)	19 (40%)/28(60%)	n.s
BMI (kg/m^2^), mean (SD)	31.2 (7.4)	29.6 (4.4)	n.s
ASA score (*n*/%)			
1:	4 (10%)	5 (11%)	n.s
2:	18 (44%)	19 (40%)	n.s
3:	18 (44%)	22 (47%)	n.s
4:	1 (2%)	1 (2%)	n.s
Preoperative X-Ray (Kellgren–Lawrence Score) (*n*/%)			
3:	11 (27%)	15 (28%)	n.s
4:	30 (73%)	34 (72%)	n.s

*ASA* American Society of Anesthesiologists, *BMI* body mass index

The study procedure followed accepted ethical, scientific, and medical standards and was conducted in compliance with recognized international standards, including the principles of the Declaration of Helsinki. We obtained informed consent from all the participants. The study protocol was approved by the local ethics committee (30–253 ex 17/18) and a current amendment (received on the 24th of April 2020).

### COVID-19 group

Patients who underwent elective primary TKA receiving an Attune TKA (DePuy Synthes, Warsaw, IN, U.S.) during the nationwide Austrian lockdown and completed a postoperative rehabilitation were examined for their eligibility and willingness to participate during their attendance. Eligible patients were followed up 6 months postoperatively. We included patients who suffered from osteoarthritis of the knee confirmed by anteroposterior and lateral radiographs (Kellgren–Lawrence Score III/IV) and high pain levels in at least two knee compartments despite conservative treatment. Patients between the ages of 18 and 80 years who had agreed to undergo primary TKA without having been assured to receive a certain type of prosthesis were eligible to be included in the trial. Exclusion criteria were: a flexion of less than 70° (*n* = 0), varus and valgus deformities over 15° (*n* = 0), known or suspected metal allergies (*n* = 0), patients who were unwilling to participate (*n* = 3) or unable to provide informed consent (*n* = 0), and patients who did not receive an Attune (*n* = 10) prosthesis.

**Fig. 1:**
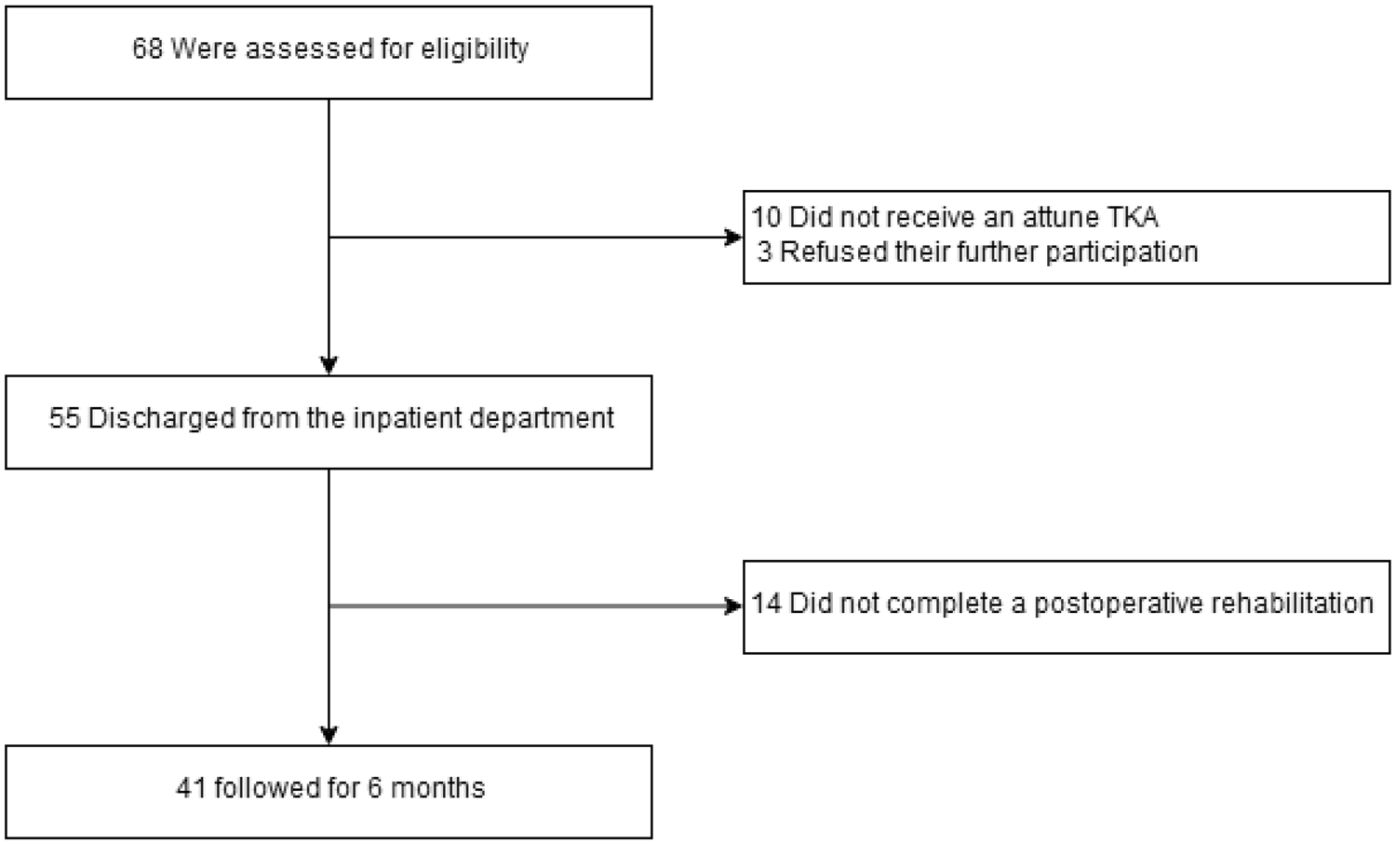
Flowchart of the included patients

Figure 1 represents a flow diagram of included patients (COVID-19 group). Fourteen patients did not complete rehabilitation after discharge from the ward. Ten patients did not receive an Attune prosthesis, and three patients refused further participation in the survey at a final follow-up visit. After a systematic search of the patients' medical records, none of the patients who were lost to follow-up or refused further participation had undergone revision surgery at the central hospital or regional partner hospitals.

All procedures were performed under the supervision of one senior knee surgeon using the same surgical technique with no patella resurfacing in each case. Femoral and tibial components were cemented (Palacos R + G, Heraeus Medical, Wehrheim, Germany) and the tibia first method with balancing of the flexion gap was carried out using a medial parapatellar approach. Every patient received a primary Attune TKA (DePuy Synthes, Warsaw, IN, U.S.), cruciate-retaining and fully cemented prothesis implanted in mechanical alignment according to Insall et al. [12] Postoperatively, the patients of the COVID-19 group followed a standardized physiotherapy protocol immediately after surgery, which consisted of full weight-bearing with crutches, active mobilization of the patient in a transverse sitting position combined with physical exercise assistance and stretching of the knee capsule, isometric quadriceps exercise, and continuous passive motion (CPM) therapy on the first postoperative day [13, 14]. However, due to the pandemic, the postoperative protocol had to be shortened in terms of hospital length of stay and was only possible under strict hygiene measures that did not allow adequate physiotherapy as it would have taken place without the pandemic situation.

### Control group

A consecutive series of 46 patients undergoing TKA using the same prosthesis in the same surgical technique by the same surgeon, who were stratified according to age, sex, preoperative clinical scores, preoperative Kellgren and Lawrence scale and preoperative range of motion (ROM), served as the control group at 6 months of follow-up in May 2019. Comparable data regarding age, BMI, Kellgren and Lawrence scores on preoperative radiography, preoperative clinical scores, and ROM are presented in Tables 1 and 2.

**Table 2:** Comparison of clinical outcome before and after the final follow-up (6 months)

	COVID (*n* = 41)	Non-COVID (*n* = 47)	*p* value
Waiting time to surgery (mean ± SD)	150.5 ± 87.9	87.6 ± 64.1	< 0.001
Hospital LOS (mean ± SD)	7.0 ± 1.1	8.6 ± 2.2	< 0.001
ROM (°) (mean ± SD)			
Preoperative	93,7 ± 21.7	91,8 ± 21.4	0.408
6 months follow-up	110.0 ± 12.8	115.1 ± 11.2	0.132
WOMAC (mean ± SD)			
Preoperative	56.1 ± 11.1	54.2 ± 14.8	0.551
6 months follow-up	29.8 ± 16.0	20.9 ± 12.1	0.001
KSKS (mean ± SD)			
Preoperative	50.4 ± 13.8	51.6 ± 12.4	0.621
6 months follow-up	76.4 ± 20.7	89.7 ± 12.2	< 0.001
KSFS (mean ± SD)			
Preoperative	45.1 ± 14.6	46.8 ± 17.6	0.941
6 months follow-up	87.4 ± 16.3	90.0 ± 11.0	0.933
VAS at rest (mean ± SD)			
Preoperative			
6 months follow-up	1.9 ± 1.7	1.7 ± 1.1	0.922
VAS at movement (mean ± SD)			
Preoperative	6.1 ± 1.9	6.4 ± 1.9	0.452
6 months follow-up	3.2 ± 2.0	2.9 ± 2.0	0.304
OKS (mean ± SD)			
Preoperative	23.6 ± 6.7	24.9 ± 8.7	0.583
6 months follow-up	34.0 ± 9.5	43.1 ± 6.0	< 0.001

*ROM* range of motion, *WOMAC* Western Ontario and McMaster Universities Osteoarthritis Index, *Ksks* Knee Society Knee Score, *KSFS* Knee Society Function Score, *VAS* visual analogue scale, *OKS* Oxford Knee Score, *LOS* length of stay, *SD* standard deviation

Patients in the control group followed the same standardized inpatient physiotherapy protocol as the COVID-19 group, but without strict hygiene measures and could stay longer in the hospital than the COVID-19 group. Our physiotherapy regime consists of passive mobilization therapy using continuous passive motion three times a week within the first 4 weeks after surgery in patients not bending their knees over 90 degrees at discharge (6 days postoperatively) and active stability exercises 3 weeks after surgery. Included patients also received a primary Attune TKA (DePuy Synthes, Warsaw, IN), cruciate-retaining and fully cemented prothesis implanted in mechanical alignment according to Insall et al. [12]. Therefore, the control group was able to follow inpatient and external physiotherapy and rehabilitation 6–8 weeks post-surgery in a rehab centre after physiotherapy.

### Physiotherapy/rehabilitation during the lockdown

The first Austrian lockdown started on the 16th of March 2020 [15]. Nationwide, from the 16th of March until the 20th of April 2020, homes could only be left for necessary professional activities, necessary purchases such as groceries or medication, assisting other people, and activities outside (alone or accompanied by people living in the same household). From the 20th of March until the 30th of April, a ban on entering rehabilitation facilities, including external physiotherapy and rehabilitation, was imposed except for the use of necessary medical rehabilitation measures following acute medical treatment [16]. On the 1st of May, the lockdown restrictions became obsolete.

Consequently, patients of the COVID-19 group had no access to external physiotherapy and rehabilitation during this time. In May 2020, external physiotherapy facilities were reopened, whereas registered physiotherapists with a private practice only gradually opened. As a result, external physiotherapy was only possible under certain conditions such as individual contact with patients and protective equipment limiting the physical exercise after discharge from May onwards. In contrast to the limitations in physiotherapy in the hospital and external institutions, the therapy programme for rehabilitation in external facilities (rehabilitation centres) was not reduced after the entry ban [17].

In other words, the COVID-19 group faced challenges during the pandemic in physiotherapy in contrast to the control group.

### Outcome measurement

The clinical status and preoperative and postoperative outcomes at 6 months were assessed using the Knee Society Knee Score (KSKS) and Knee Society Function Score (KSFS) [8], the Western Ontario and McMaster Universities Osteoarthritis Index (WOMAC) [9], the visual analogue scale (VAS) for pain both at rest and in motion, the Oxford Knee Score (OKS) [10], and the range of motion (ROM). The ROM was measured with a double-armed goniometer. Lengths of hospital stay and the waiting time to surgery (in days) were also determined.

### Statistical analysis

The data were analysed by SPSS Version 23.0 (IBM Corporation, New York, USA). Descriptive statistics for continuous variables were reported as the mean and standard deviation (SD). Categorical variables were reported as count and proportions. For comparisons of categorical variables, the Chi-square exact test was used. The data were tested for normality using the Kolmogorov–Smirnov test, which revealed a parametric distribution for the BMI and age and a non-parametric distribution for all clinical scores, ROM, hospital LOS, and waiting time (in days) to surgery. Group differences were observed through the *t* test and the Mann–Whitney *U* test. Statistical significance was achieved at a *p* value < 0.05. Post hoc power analysis was calculated according to Hoenig and Heisey for the magnitude of differences in all compared scores and parameters. The selected sample size per group was sufficient for the analysis.

According to Hoenig and Heisey, the observed power was calculated to be greater than 80% regarding the difference in the included clinical scores and analysed parameters, revealing a *p* value of < 0.01 [18]. The magnitude of the difference between both groups reached a post hoc power greater than 80% (according to Hoenig and Heisey).

## Results

In Table 2, the comparison of clinical outcomes, length of stay (LOS), and waiting time to surgery is summarized. At the 6 months follow-up, we could observe significantly inferior clinical outcomes in the COVID-19 group compared to the control group in WOMAC (29.8 vs. 20.9; *p* = 0.001), KSKS (76.4 vs. 89.7; *p* < 0.001), and OKS (34.0 vs. 43.1; *p* < 0.001). No difference was observed in KSFS (87.4 vs. 90.0; *p* = 0.933), ROM (110.0 vs. 115.1; *p* = 0.132), VAS at rest (1.9 vs. 1.7; *p* = 0.9.22) and during exercise (3.2 vs. 2.9; *p* = 0.304) at the final evaluation. In the final follow-up cohorts, no minor or major complications that required further surgical treatment were observed.

The COVID-19 group had significantly shorter hospital stays (7.0 vs. 8.6; *p* < 0.001) and had to wait almost twice as long (150.5 vs. 87.6; *p* < 0.001) for their surgery appointment.

## Discussion

The most important findings were statistically significant clinical differences in functional outcome parameters between the groups. The COVID-19 group showed significantly worse results in terms of WOMAC (29.8 vs. 20.9; *p* = 0.001), KSKS (76.4 vs. 89.7; *p* < 0.001), and OKS (34.0 vs. 43.1; *p* < 0.001).

The current study's findings indicate that the current pandemic negatively affected patients who underwent elective primary TKA during the lockdown, resulting in significantly inferior clinical outcomes at a minimum follow-up of 6 months compared to a control group. These differences are strongly linked to the restrictions during the first Austrian lockdown that led to missing physiotherapy. When comparing these two groups, the functional results are also dependent on the remaining limitations brought about by the pandemic. The postoperative inpatient physiotherapy of the COVID-19 group was shortened and only possible under strict hygiene measures that did not allow adequate therapy as it would have taken place without the pandemic. After the postoperative inpatient management, the COVID-19 group could not go to any external facilities for physiotherapy during the lockdown from the 20th of March until the 30th of April [6]. As a result, only exercises learnt from inpatient physiotherapy—such as climbing stairs and walking exercises in the horizontal at home—were practised at this time, resulting in inferior clinical outcomes 6 months after TKA.

Regarding external rehabilitation, the entry ban on rehabilitation facilities was in effect up until the 30th of April 2020 [6]. In general, external rehabilitation after TKA starts 6–8 weeks post-surgery. Also, the waiting period for rehabilitation entry and the therapy programme in rehabilitations centres for patients after TKA was not different from that before COVID-19. Only hygiene measures (e.g. single room, no group activities, no social evenings) were implemented, not changing the therapy programme itself. Giesinger et al. determined a definition of ‘treatment success’after TKA. Based on their study, the respective thresholds for the WOMAC osteoarthritis index and the Knee Society Score (KSS) at 2 months after TKA are 29.5 (WOMAC Total), 75.5 KSKS, and 42.5 KSFS [19]. Comparing the cutoff values 2 months after TKA, as described by Giesinger et al. [20], with our results 6 months post-surgery, both our study groups reached the thresholds.

Moorthy et al. [22] compared gap balancing and measured resection; our comparison group had a worse clinical outcome. Studies that describe short-term results on ROM had better results than our COVID-19 group [23, 24]. Although the differences in ROM between our two groups were not statistically significantly different, our findings might show a potential significant clinical difference.

The literature has repeatedly described the importance of physiotherapeutic exercise, both inpatient and outpatient rehabilitation [25], which leads to a significant improvement in the clinical outcome. [11, 25–27]. According to the findings of den Hertog et al., a comprehensive fast-track rehabilitation protocol leads to a statistically significant improved functional outcome in the KSS and WOMAC, having a sustainable effect on the mid-term outcome 3–12 months after surgery [8]. Moreover, Kondo et al. observed significantly greater improvements in the total WOMAC score, pain, and function 3 weeks after TKA when isometric quadriceps exercise in usual rehabilitation was involved [18].

Despite active physiotherapy, the meta-analysis data of Brosseau et al. show significant improvements when continuous passive motion (CPM) is combined with physiotherapy at 2 weeks after TKA, resulting in a significantly increased active knee flexion [17].

These findings suggest that sufficient physiotherapy is of great relevance and therefore essential to attain excellent early functional outcomes following TKA. However, it also reflects the essential role of orthopaedic care in the healthcare system and the necessity of keeping trauma and orthopaedic surgery in business, even in a reduced manner.

The observed increase in preoperative waiting time of the COVID-19 group (150.5 vs. 87.6; *p* < 0.001) is in accordance with the findings of a study that examined the impact of the COVID-19 pandemic on the LOS in patients following total hip and knee arthroplasty [28]. In contrast to the mentioned study [28], the length of hospital stay of our COVID-19 group was significantly shorter (7.0 vs. 8.6; *p* < 0.001) than that of the control group. We interpret this discrepancy to be as a result of reduced infrastructural capacities. Waiting time is structured in two groups at our department. Patients with osteonecrosis or after implant failure or still in their profession undergo surgery within 6–8 weeks after their first visit to our outpatient’s clinic. Other patients undergo surgery according to the actual waiting list. During the COVID-19 pandemic, patients with ASA status of 4 could not undergo surgery as there was lack of capacity at our intensive care unit. These factors all contributed to the presented waiting list.

In the COVID-19 group, eight coronavirus disease cases were reported, but all were mild. No limitation as described in Wang et. al. was expected. [29] Thus, postoperative COVID-19 symptoms and associated respiratory complications had no effect on the outcome of our work.

The study presents different limitations. First, a retrospective study has a low level of evidence in general, and level I or II studies might have been optional concerning the included number of cases. Results should be interpreted accordingly. Therefore, the applicability of our findings is limited, and the impact on the orthopaedic surgery sector needs to be investigated in broader studies. However, as the COVID-19 pandemic and its lockdown were not planned, our setting was a reasonable approach.

We want to underline the following aspects when revealing our conclusions. All TKAs were performed under the supervision of one senior knee surgeon and in a standardized setting at a single institution. Even though this data can be assumed to be a valid representation of the distribution of elective total knee arthroplasties within an orthopaedic centre, the impact on the overall orthopaedic care needs to be interpreted with caution. Due to the restrictions, patients were harmed in many ways, such as restricted movement and social interactions—parameters we did not evaluate in the present study—that might have contributed to worse outcomes observed in the COVID-19 group. In addition, possible worsening of the mental state (e.g. increased distress) [30] in the COVID-19 group compared to the control group, which we did not examine, may also have affected the results.

This retrospective comparative study provides additional and valuable information on the early clinical outcome after total knee arthroplasty in a nationwide lockdown despite the mentioned limitations. The results emphasize the need for a prolonged follow-up evaluation.

## Conclusion

The early clinical outcome after elective total knee arthroplasty is affected by the limited options for physiotherapy after discharge, which were caused by the lockdown during the COVID-19 pandemic. We believe that standardized protocols for physiotherapy will improve clinical outcomes for TKA in the event of future lockdowns and underline the importance of appropriate postoperative care during this pandemic.
